# Disease asymmetry and hyperautofluorescent ring shape in retinitis pigmentosa patients

**DOI:** 10.1038/s41598-020-60137-9

**Published:** 2020-02-25

**Authors:** Ruben Jauregui, Lawrence Chan, Jin Kyun Oh, Ahra Cho, Janet R. Sparrow, Stephen H. Tsang

**Affiliations:** 1 Edward S. Harkness Eye Institute, Columbia University Medical Center, New York, NY USA; 2 Jonas Children’s Vision Care and Bernard & Shirlee Brown Glaucoma Laboratory, New York, NY USA; 3 Weill Cornell Medical College, New York, NY USA; 4 Department of Ophthalmology, University of California San Francisco, San Francisco, CA USA; 5 State University of New York at Downstate Medical Center, Brooklyn, NY USA; 6 Department of Pathology & Cell Biology, Columbia University, New York, NY USA

**Keywords:** Genetics research, Molecular medicine

## Abstract

Retinitis pigmentosa (RP) is described as a bilateral disease with inter-eye symmetry that presents on short-wavelength fundus autofluorescence (SW-AF) imaging with hyperautofluorescent (hyperAF) rings with an ellipsoid shape and regular borders. Nevertheless, both asymmetry and irregular ring morphologies are also observed. In this retrospective study of 168 RP patients, we characterize the degree of inter-eye asymmetry and frequency of irregular hyperAF ring morphologies according to mode of inheritance and disease-causing gene by using SW-AF imaging and spectral-domain optical coherence tomography (SD-OCT) scans. We observed that from 336 eyes, 290 (86%) presented with regular hyperAF rings and 46 (14%) presented with irregular shapes. From the 168 patients, 23 (14%) presented with asymmetric disease, with 16 (70%) of these patients also presenting with irregular ring shapes. Patients with autosomal dominant RP (adRP) had the highest proportion of irregular ring shapes (21%) and disease asymmetry (23%) in comparison to other modes of inheritance. Furthermore, both *RP1* and *RHO*-adRP had the highest proportions of both disease asymmetry and irregular ring morphology. Our results suggest that in patients presenting with either irregular ring shapes or asymmetric disease, emphasis should be placed in targeted gene sequencing of genes known to cause adRP, such as *RHO* and *RP1*.

## Introduction

Retinitis pigmentosa (RP) is a group of heterogenous rod-cone retinal dystrophies caused at a cellular level by the degeneration of photoreceptors^1,2^. In most cases, the genetic defect is exclusive to the rods, whose degeneration leads to secondary cone death^2,3^. Patients clinically present with night blindness, constricted visual fields, and an eventual decrease in central vision that ultimately leads to blindness in the late stages. Other clinical findings on examination of the posterior pole include intraretinal pigment migration associated with retinal pigment epithelium (RPE) thinning, attenuated blood vessels, and pallor of the optic nerve^4^. The prevalence of RP is estimated to be 1 in 4,000, and the disease can be inherited in an autosomal dominant (30–40%), autosomal recessive (50–60%), or X-linked (5–15%) manner^1^.

RP is often described as a bilateral disease that presents and progresses symmetrically between both eyes. Exceptions are rare, but unilateral RP may account for 5% of the total population of RP patients^5^. Other pathologies that have been reported to cause unilateral pigmentary retinopathy include infection, inflammation, and trauma^6–9^. Nevertheless, few studies exist that analyze the degree of disease asymmetry among RP patients. An early study by Biro *et al*. suggested that a feature of RP is the symmetrical development of pigmentation, while a study by Massof *et al*. reported a high degree of symmetry as measured by visual fields^10,11^. More recent studies by Sujirakul *et al*. and Fakin *et al*. have also reported a high degree of symmetry, although asymmetry does occur^12,13^. Nevertheless, these studies did not explore the role that mode of inheritance and specific disease-causing genes play in affecting disease asymmetry in RP.

In this study, we explore disease asymmetry between fellow eyes in a cohort of RP patients by measuring the dimensions of the hyperautofluorescent (hyperAF) ring on short-wavelength fundus autofluorescence (SW-AF) imaging and the width of the ellipsoid zone (EZ) line on spectral-domain optical coherence tomography (SD-OCT) scans, parameters that are often used to track disease progression in RP^14,15^. In addition, we explore the degree of asymmetry and its relationship to mode of inheritance, patient genotype, and shape of the hyperAF ring.

## Methods

### Patients and clinical examination

The study procedures were defined and informed patient consent was obtained as outlined by the protocol #AAAR0284 approved by the Institutional Review Board at Columbia University Medical Center. The study is adherent to the tenets of the Declaration of Helsinki. The data presented in this study, including images and genetic testing results, is not identifiable to individual patients. A retrospective review of patients with a clinical diagnosis of RP by an inherited retinal disease specialist (SHT) at the Department of Ophthalmology at Columbia University was conducted. The clinical diagnosis was based on presenting symptoms, family history, fundus examination, and subsequently supported by clinical imaging, full-field electroretinography (ffERG), and/or genetic testing. Ophthalmic examinations included a slit-lamp and dilated funduscopic examination, best-corrected visual acuity (BCVA), short-wavelength fundus autofluorescence (SW-AF, 488 nm excitation), and spectral domain optical coherence tomography (SD-OCT). The inclusion criteria for this study were the diagnosis of RP and genetic characterization of the disease, while the exclusion criteria precluded monocular patients or those without SD-OCT or SW-AF imaging.

Imaging across all modalities was conducted after pupil dilation (>7 mm) with phenylephrine hydrochloride (2.5%) and tropicamide (1%). Horizontal foveal SD-OCT scans and SW-AF (488 nm excitation) were acquired with the Spectralis HRA + OCT (Heidelberg Engineering, Heidelberg, Germany). The SW-AF images were acquired with either a 55 or 30-degree field of view such that the entire hyperAF ring could be appreciated within the image.

### Image analysis

The SW-AF images from the patients that met the inclusion criteria for the study were analyzed independently by two different graders (RJ and LC). The hyperAF rings were grouped into two categories based on morphology: 1) regular and 2) irregular. Regular rings were defined as closed rings with an ellipsoid/round shape and regular borders, while irregular rings included any ring morphologies that deviated from the above, including open rings, closed rings with irregular borders, and closed rings with non-ellipsoid shapes. Measurements of the horizontal and vertical diameters of the hyperautofluorescent ring, along with the width of the ellipsoid zone (EZ) line from the SD-OCT scans, were performed by the two graders on closed rings only, as not all of these parameters were always well-defined in open rings. A total of 151 patients (302 eyes) from the total cohort of 168 presented with closed rings. The measurements on both eyes of each patient were performed using a built-in measurement tool in the Spectralis HRA + OCT software. The horizontal diameter was defined as the longest distance between the nasal and temporal borders of the ring, while the vertical diameter was perpendicular to the defined horizontal diameter. The external boundary of the ring, which is better defined than the internal boundary, was used as the borderline for the diameter measurements.

### Statistical analysis

The statistical analyses were performed using Stata 12.1 (StataCorp, College Station, Texas, USA) software. The Pearson correlation was calculated for the measurements of both independent graders. Given the high correlation between the two graders (r = 0.99, p < 0.001 for all parameters measured), the average of the two values obtained from the graders was calculated and used for subsequent analysis. For each parameter, the difference between both eyes was calculated and descriptive statistics for the horizontal, vertical diameters, and EZ line width were calculated (see Supplementary Table S1). Given the categorical nature of our data, chi-squared tests were used to compare disease asymmetry and ring morphology among the different modes of inheritance and to assess for an association between categorical variables.

### Asymmetry analysis

Patients were defined to have asymmetric disease if they exhibited a difference greater than the 95^th^ percentile cut-off in one or more of the parameters. Additionally, if patients exhibited a different ring morphology in each eye, they were also considered to have asymmetric disease. For patients with open rings, only the best-defined parameter was considered in the analysis. Patients were analyzed as an entire cohort and as sub-cohorts based on mode of inheritance of the disease (autosomal dominant, autosomal recessive, and X-linked recessive). Patients were also divided into sub-cohorts based on the identified disease-causing gene, and only those cohorts with ten or more patients were analyzed.

## Results

### Patients

In total, 168 patients with RP were analyzed for this study. Among the 168 patients, 57 (34%) presented with adRP, 100 (60%) with arRP, and 11 (6%) with XLRP. From the arRP patient cohort, 22 patients presented with syndromic disease: 6 with Usher syndrome type I, 13 with Usher type II, 2 with Usher type III, and 1 with Bardet-Biedl syndrome. The average age of the patients was 40 years old, which was similar to the average age of the sub-cohorts, except for XLRP, where the average age was 25 years. The most common disease-causing gene was *RHO* for adRP (37%), *USH2A* for arRP (42%), and *RPGR* for XLRP (100%). Patient demographics and genetic characterization are summarized in Table 1 (for more complete demographic and genetic characterization, see Supplementary Table S2).

**Table 1:** Demographics and genetic characterization for the cohort of patients.

Patient Cohorts	N (%)	Age During Visit
RP total	168 (100)	40.4 ± 18.9
adRP	57 (34)	42.8 ± 17.9
arRP	100 (60)	40.7 ± 19.1
XLRP	11 (6)	24.6 ± 15.7
**Forms of RP**	**N**	**Genes with disease-causing variants (N)**
adRP	57	*RHO* (21), *RP1* (12), *PRPF31* (10), *PRPF8* (4), *SNRNP200* (3), *KLHL7* (3), *GUCA1B* (1), *NRL* (1), *PRPF3* (1), *PRPH2* (1)
Non-syndromic arRP	78	*USH2A* (31), *EYS* (12), *PDE6B* (7), *CNGB1* (4), *MAK* (4), *KIZ* (3), *PDE6A* (3), *DHDDS* (2) *FAM161A* (2), *MERTK* (2), *C21ORF2* (1), *PCDH21* (1), *PROM1* (1), *REEP6* (1), *SCAPER* (1), *TULP1* (1), *RP1* (1), *SPATA7* (1)
Syndromic arRP	22	
USH 1	6	*MYO7A* (5), *PCDH15* (1)
USH 2	13	*USH2A* (11), *GPR98* (2)
USH 3	2	*CLRN1* (2)
BBS	1	*BBS1* (1)
XLRP	11	*RPGR* (11)

Data are summarized as mean ± standard deviation where appropriate. BBS = Bardet-Biedl syndrome; N = number; RP = retinitis pigmentosa; arRP = autosomal recessive; adRP = autosomal dominant; USH = Usher syndrome; XLRP = X-linked recessive.

### Morphology of the hyperautofluorescent rings

**Figure 1:**
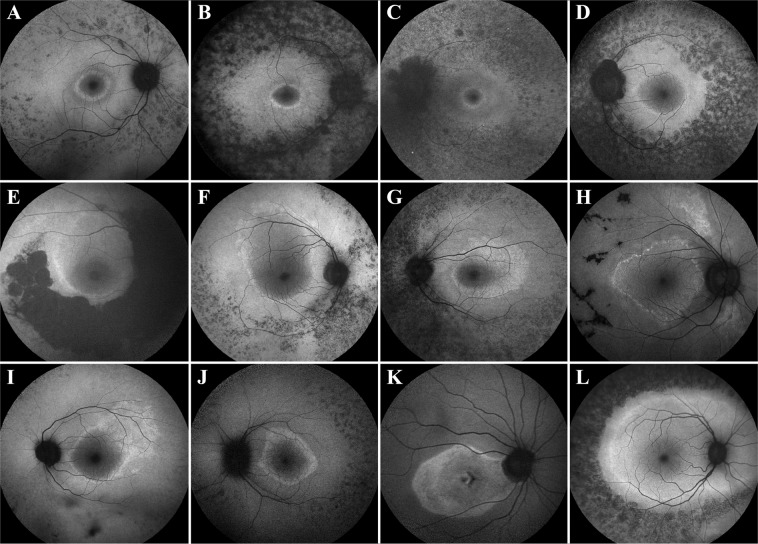
Regular and irregular hyperautofluorescent rings on patients with retinitis pigmentosa. Short-wavelength fundus autofluorescence (SW-AF) imaging reveals hyperautofluorescent rings with the regular, ellipsoid shape in the majority of patients, as seen in Patients. (**A**–**D**) A minority of patients, however, can present with irregular ring shapes in a variety of forms as observed in Patients. (**E**–**L**) Disease-causing genes for these patients are *USH2A* (**A**,**B**,**D**,**J** and **L**), *PDE6B* (**C**), *SNRNP200* (**E**), *MAK* (**F**,**I**), *PRPF8* (**G**), *BBS1* (**H**), and *SPATA7* (**K**).

When analyzing the morphology of the hyperAF rings, the majority of patients presented with regular shapes (86% of eyes), while the rest presented with irregular shapes (Fig. 1). When segregating the patients by mode of inheritance, we observed that irregular ring morphology is more common in the autosomal dominant (21%) as compared to the autosomal recessive (10%, P = 0.007) and X-linked (9%, P = 0.191) forms. We also analyzed the occurrence of regular and irregular ring shapes by segregating patients into cohorts by disease-causing gene. We found that there is an association between the disease-causing gene and whether the ring presents with regular or irregular morphology (P = 0.004). We observed that disease caused by *EYS*, *RP1*, and *RHO* presented with the highest proportions of irregular rings (29%, 23%, and 21%, respectively), whereas *PDE6A/B*, *USH2A*, and *RPGR* have the highest proportions of regular rings (100%, 96%, and 91%, respectively). This information is summarized in Table 2.

**Table 2:** Characterization of the shapes of the hyperautofluorescent rings in retinitis pigmentosa patients organized in sub-cohorts by mode of inheritance and disease-causing gene.

Patient Cohorts	N	Eyes (N)	Hyperautofluorescent Ring Shape	
			Regular (%)	Irregular (%)
Total	168	336	290 (86%)	46 (14%)
adRP	57	114	90 (79%)	24 (21%)
arRP	100	200	180 (90%)	20 (10%)
XLRP	11	22	20 (91%)	2 (9%)
**Patient Cohorts by Disease-causing Gene**				
*EYS*	12	24	17 (71%)	7 (29%)
*PDE6A/B*	10	20	20 (100%)	0 (0%)
*PRPF31*	10	20	17 (85%)	3 (15%)
*RHO*	21	42	33 (79%)	9 (21%)
*RP1*	13	26	20 (77%)	6 (23%)
*RPGR*	11	22	20 (91%)	2 (9%)
*USH2A*	42	84	80 (96%)	4 (4%)

RP = retinitis pigmentosa; arRP = autosomal recessive; adRP = autosomal dominant; XLRP = X-linked recessive.

### Patients with disease asymmetry

**Figure 2:**
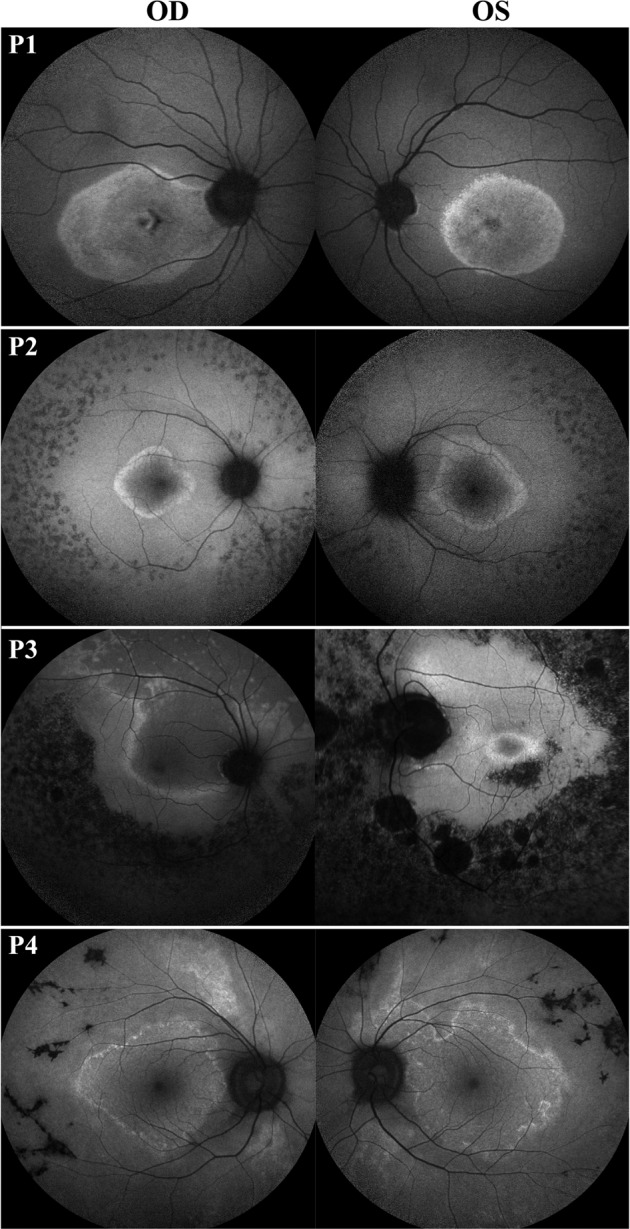
Asymmetric disease on observed in retinitis pigmentosa patients. Short-wavelength fundus autofluorescence (SW-AF) imaging of patients with disease asymmetry between both eyes. Disease-causing genes for these patients are *SPATA7* (Patient 1), *USH2A* (Patient 2), *EYS* (Patient 3), and *BBS1* (Patient 4).

From our patient cohort, we found that 23 patients (14%) presented with asymmetric disease (Fig. 2). Autosomal dominant RP presented with the highest proportion of asymmetric disease (23%) as compared to autosomal recessive (9%, P = 0.017) and X-linked (9%, P = 0.303). Furthermore, the majority of patients with asymmetric disease presented with irregular ring shapes (70%, P < 0.001). When segregating by disease-causing gene, we observed that *RP1*-adRP, *RHO*-adRP, and *PRPF31*-adRP presented with the highest proportion of patients with asymmetric disease (31%, 24%, and 10%, respectively), whereas *PDE6A/B*-arRP and *USH2A*-arRP presented with the lowest proportion (0% and 7%, respectively). This information is summarized in Table 3.

**Table 3:** Characterization of disease asymmetry in retinitis pigmentosa patients organized in sub-cohorts by mode of inheritance and disease-causing gene.

Patient Cohorts	N	Patients with Asymmetric Disease (%)	Patients with Asymmetric Disease and Irregular Ring Shape
Total	168	23 (14%)	16 (70%)
adRP	57	13 (23%)	9 (69%)
arRP	100	9 (9%)	6 (67%)
XLRP	11	1 (9%)	1 (100%)
**Patient Cohorts by Disease-causing Gene**			
*EYS*	12	1 (8%)	1 (100%)
*PDE6A/B*	10	0 (0%)	0 (0%)
*PRPF31*	10	1 (10%)	1 (100%)
*RHO*	21	5 (24%)	2 (40%)
*RP1*	13	4 (31%)	4 (100%)
*RPGR*	11	1 (9%)	1 (100%)
*USH2A*	42	3 (7%)	0 (100%)

N = number; RP = retinitis pigmentosa; arRP = autosomal recessive; adRP = autosomal dominant; XLRP = X-linked recessive.

## Discussion

With the recent advances of ocular gene therapy as a promising treatment modality for retinal dystrophies, it is important to study the level of asymmetry in these diseases, as the contralateral eye is often used as a control for the treatment eye due to the assumption of disease symmetry^16–19^. Although some studies have studied asymmetry in RP, they are either limited to a certain population of RP patients or are lacking in genetic characterization, a crucial aspect in a patient’s diagnosis due to the mutation-specific nature of gene therapy. In a study by Fakin *et al*., for example, 54 patients with Usher Syndrome type I and II were characterized with SD-OCT and SW-AF, and they report asymmetry in 10% of their patients. A different study by Sujirakul *et al*. used SW-AF to measure the horizontal and vertical diameters of the hyperAF ring, and they reported asymmetry in approximately 14% of their patients^12^. Nevertheless, genetic characterization was only available for 30 out of the 88 (34%) patients they analyzed^12^. In our study, we observed asymmetry in 14% of our patients, which is similar to what the above two studies reported. Moreover, the complete genetic characterization of our patient cohort allowed us to not only analyze asymmetry in RP, but to also correlate asymmetry with mode of inheritance.

We observed that adRP presents with both higher proportions of patients with irregular hyperAF ring shapes (24%) and asymmetry (23%), as compared to arRP (10% and 9%, respectively) and XLRP (2% and 9%, respectively). We theorize that this higher proportion of disease asymmetry in adRP might be related to genetic factors and variable expressivity of the diseased allele. As compared with other forms of inheritance, adRP is known to frequently present with variations in expressivity, and multiple studies have analyzed variable expressivity and incomplete penetrance in genes that cause adRP such as *PRPF8* and *PRPF31*^2,20,21^. A similar study to ours analyzed asymmetry in the disease progression of Stargardt disease, where the authors report that lower inter-eye correlations are more likely to be found on late-onset Stargardt disease^22^. Similar to adRP as compared to arRP or XLRP, late-onset Stargardt is milder than the other forms of the disease, such as early-onset^23^. Thus, these results suggest that disease asymmetry might be associated with mild disease severity. Of note, despite observing a higher proportion of patients with asymmetry and irregular AF ring shapes in adRP as compared to XLRP, we did not observe a statistically significant difference when comparing the two. We believe that this is due to the low number of XLRP patients in our cohort, as asymmetry is uncommon in RP and our cohort only contains 11 (6%) XLRP patients.

We categorized the hyperAF rings into two groups: regular and irregular ring shapes. The vast majority of patients presented with regularly shaped rings (86% of eyes analyzed). Similar to the analysis of disease asymmetry, adRP also presented with the greatest proportion of rings with irregular shapes. Currently, studies using quantitative autofluorescence (qAF) suggest that the mechanism for ring formation involves accelerated bisretinoid formation in actively degenerating receptor cells^24^. Clinically, we also know how the hyperAF ring relates to a patient’s vision. The signal for SW-AF (488 nm excitation) is derived mostly from RPE lipofuscin, which is formed in the photoreceptors as a byproduct of all-*trans*-retinal reactions^25–27^. The inner border of the hyperAF in RP patients corresponds to the lateral end of the EZ line on SD-OCT, and as disease progresses, the EZ line shortens along with constriction of the hyperAF ring^24,28^. Previous studies have shown that the point at which the EZ line disappears corresponds to the edge of the patient’s visual field and marks the boundary between healthy and unhealthy retina^28–30^. The results from these studies help us to conclude that not only is asymmetry observed in SW-AF and SD-OCT images, but also in functional vision parameters such as visual fields. Visual acuity, however, should not be affected by the asymmetric process, as RP starts on the periphery and affects central vision during the later stages of the disease. In fact, we observed no difference in the degree of asymmetry in visual acuity between fellow eyes in our cohort of patients with asymmetric disease as compared to those with symmetric disease (P = 0.255). The presentation of patients with asymmetric RP was also similar to patients with symmetric RP in regards to age of disease onset and severity of disease. It has been widely reported that XLRP is more severe than adRP^31^. This was also observed in our patient cohort, regardless of whether the patient presented with asymmetric disease or not. As expected, patients with *RPGR*-XLRP, for example, presented with more aggressive disease and earlier onset as compared to patients with *RHO*-adRP.

In addition to mode of inheritance, we also decided to analyze disease asymmetry and ring morphology when stratifying by the disease-causing gene. From our patient cohort, we observe that although every gene exhibits a regular ring shape more frequently, genes like *EYS*, *RP1*, and *RHO* have a higher proportion of patients with irregular ring morphology (29%, 23%, and 21% of eyes, respectively). This is in contrast to genes where irregular ring morphologies were observed infrequently, such as the *PDE6A/B* family, *USH2A*, and *RPGR* (0%, 4%, and 9%, respectively). Similarly, we observed disease asymmetry proportions were the highest in *RP1* (31%) and *RHO* (24%), which generally have milder presentation than disease caused by the *PDE6A/B* family, *USH2A*, and *RPGR*.

**Figure 3:**
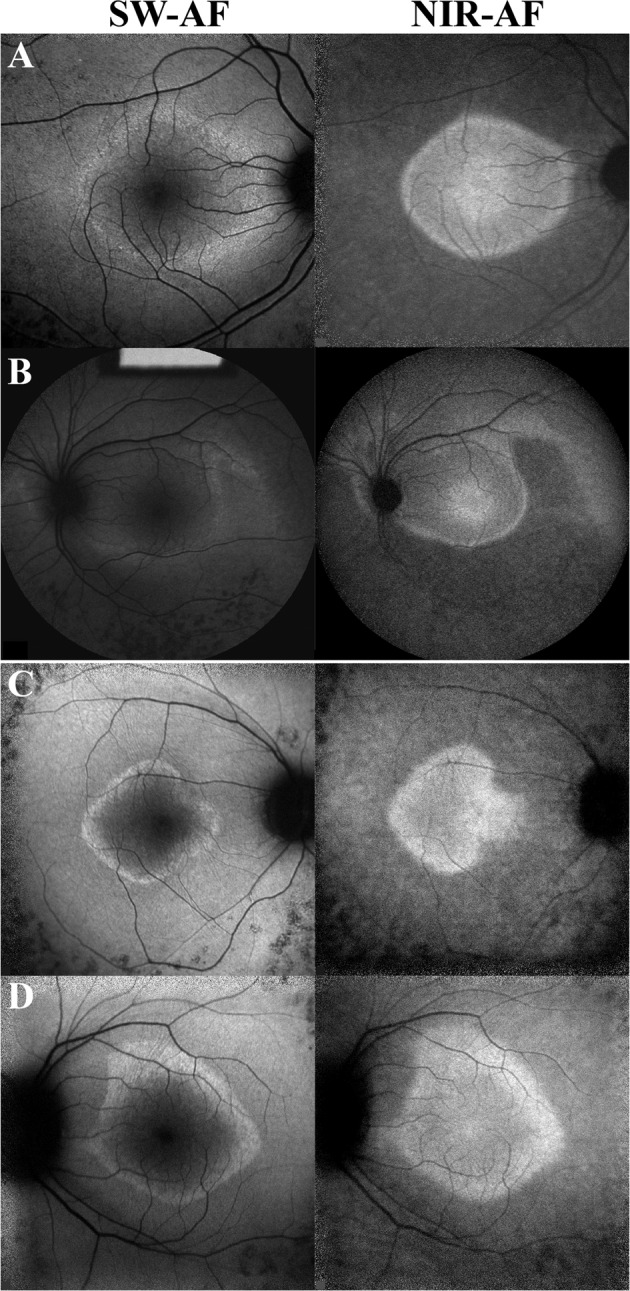
Asymmetric disease with irregular rings as observed in short-wavelength (SW-AF) and near-infrared (NIR-AF) fundus autofluorescence. Patient 1 presented with RHO-autosomal dominant retinitis pigmentosa (**A**,**B**) and Patient 2 presented with USH2A-autosomal recessive retinitis pigmentosa. (**C**,**D**) Disease asymmetry and irregular rings are observed in both patients on SW-AF and NIR-AF images.

We were able to characterize asymmetry based on the hyperAF rings observed in SW-AF imaging. Similarly, hyperAF rings are also observed in near-infrared autofluorescence imaging (NIR-AF)^14,32^. Previous studies from our group have reported that the hyperAF rings appear larger in SW-AF imaging and that similar rates of disease progression are observed in both modalities^14,32^. In the patient cohort we present for this study, only a few patients had been imaged with both modalities. Yet, in those patients, we were able to observe disease asymmetry in both NIR-AF and SW-AF imaging (Fig. 3). Future studies with larger cohorts of patients with both SW-AF and NIR-AF imaging should analyze the extent to which NIR-AF imaging demonstrates asymmetry as compared to SW-AF.

In conclusion, our study suggests genotype-phenotype correlations that can help the clinician in the diagnosis and management of RP patients. Based on our results, there is a relationship between symmetry of disease and ring morphology. Thus, if a patient presents with asymmetric disease or irregular rings on SW-AF, a diagnosis of adRP is more likely. Among the different methods of genetic sequencing, such as clinical exome or clinical genome, targeted sequencing has greater availability to patients, as its price is lower and results are obtained in less time. If either clinical exome or clinical genome sequencing cannot be obtained for a patient with asymmetric disease, emphasis should be placed in targeted sequencing of genes known to cause adRP, such as *RP1* and *RHO*. Furthermore, given the higher likelihood of adRP disease, emphasis should be placed in screening other family members. Our results also beget the important question of whether disease progression is asymmetric between fellow eyes in those patients with asymmetric disease, which future studies should address.

## Supplementary information

Supplmentary Table 1 and 2.

## Data Availability

The datasets generated during and/or analyzed during the current study are available from the corresponding author on reasonable request.
