# Supplementary material for: Disease asymmetry and hyperautofluorescent ring shape in retinitis pigmentosa patients
Source: Sci Rep. 2020 Feb 25;10:3364. doi: 10.1038/s41598-020-60137-9 (PMC7042348; doi:10.1038/s41598-020-60137-9)
Supplement: Supplementary file 1 — Supplmentary Table 1 and 2. [file 41598_2020_60137_MOESM1_ESM.pdf]

Disease asymmetry and hyperautofluorescent ring shape in retinitis pigmentosa patients

Ruben Jauregui [1,2,3], Lawrence Chan [1,2,4], Jin Kyun Oh [1,2,5], Ahra Cho [1,2], Janet R. Sparrow [1,2,6], Stephen H. Tsang [1,2,6, §].

[1] Edward S. Harkness Eye Institute, Columbia University Medical Center, New York, NY, USA
[2] Jonas Children's Vision Care and Bernard & Shirlee Brown Glaucoma Laboratory, New York, NY, USA
[3] Weill Cornell Medical College, New York, NY, USA
[4] Department of Ophthalmology, University of California San Francisco, San Francisco, CA, USA
[5] State University of New York at Downstate Medical Center, Brooklyn, NY, USA
[6] Department of Pathology & Cell Biology, Columbia University, New York, NY, USA
[§] Corresponding author

Running title: Asymmetry and ring shape in retinitis pigmentosa

Address Correspondence: Stephen H. Tsang, MD, PhD, Harkness Eye Institute, Columbia University Medical Center, 635 West 165th Street, Box 212, New York, NY 10032, Phone: (212) 342-1189 / Fax: 212-305-4987 / Email: sht2@cumc.columbia.edu

Supplementary Table 1. Descriptive statistics for the parameters measured in the cohort of retinitis pigmentosa patients.

	Patients (N)	Eyes (N)	Mean ± SE	Median	95_{th} Quantile	Min	Max
**EZ line width (μm)**	151	302	203 ± 24	129	619	2	2777
**Horizontal Diameter (μm)**	151	302	224 ± 31	128	719	0	4012
**Vertical Diameter (μm)**	151	302	185 ± 19	108	585	2	1476

Data are summarized as mean ± standard error (SE). RP = retinitis pigmentosa; arRP = autosomal recessive; adRP = autosomal dominant; XLRP = X-linked recessive; EZ = ellipsoid; Min = minimum; Max = maximum; N = number.

Supplementary Table 2. Complete demographic and genetic characterization of the patient cohort described in the study.

ID	Sex	Ethnicity	Age* (yr)	Disease	Gene	Pathogenic Variants			
						Allele 1		Allele 2	
						cDNA	Protein	cDNA	Protein
1	M	Caucasian	31	ADRP	GUCA1B	c.153_155del	p.D51del	n/a	n/a
2	F	Caucasian	36	ADRP	KLHL7	c.433A>T	p.N145Y	n/a	n/a
3	F	Asian	38	ADRP	KLHL7	c.433A>G	p.N145D	n/a	n/a
4	M	Other	67	ADRP	KLHL7	c.472T>C	p.C158R	n/a	n/a
5	M	Caucasian	20	ADRP	NRL	c.149C>T	p.S50L	n/a	n/a
6	M	Caucasian	31	ADRP	PRPF3	c.1007A>G	p.E336G	n/a	n/a
7	F	Caucasian	22	ADRP	PRPF31	c.383T>A	p.L128X	n/a	n/a
8	F	Caucasian	83	ADRP	PRPF31	c.1066G>A	p.G356S	n/a	n/a
9	F	Caucasian	15	ADRP	PRPF31	c.856-2A>G	p.?	n/a	n/a
10	F	Caucasian	48	ADRP	PRPF31	c.1222C>T	p.R408W	n/a	n/a
11	F	Caucasian	49	ADRP	PRPF31	c.528-39_531del	p.?	n/a	n/a
12	F	Caucasian	12	ADRP	PRPF31	c.528-39_531del	p.?	n/a	n/a
13	F	Hispanic	24	ADRP	PRPF31	c.322+4_322+7del	p.?	n/a	n/a
14	F	Hispanic	22	ADRP	PRPF31	c.322+4_322+7del	p.?	n/a	n/a
15	F	Hispanic	37	ADRP	PRPF31	c.420+6C>T)	p.?	n/a	n/a
16	M	Hispanic	55	ADRP	PRPF31	c.872C>T	p.Ala291Val	n/a	n/a
17	M	Caucasian	38	ADRP	PRPF8	c.6912C>G	p.Phe2304Leu	n/a	n/a
18	F	Asian	16	ADRP	PRPF8	c.5804G>A	p.R1935M	n/a	n/a
19	F	Hispanic	58	ADRP	PRPF8	c.6947_6962del	p.N2316RfsX38	n/a	n/a
20	M	Caucasian	64	ADRP	PRPF8	c.6991delG	p.E2331fs	n/a	n/a
21	M	Other	66	ADRP	PRPH2	c.652T>C	p.S218P	n/a	n/a
22	M	Caucasian	69	ADRP	RHO	c.946delT	p.C316AfsX44	n/a	n/a
23	F	Caucasian	43	ADRP	RHO	c.328T>C	p.C110R	n/a	n/a
24	F	Caucasian	15	ADRP	RHO	c.328T>C	p.C110R	n/a	n/a
25	F	Caucasian	23	ADRP	RHO	c.937-27-19del	p.?	n/a	n/a
26	M	Caucasian	15	ADRP	RHO	c.568G>A	p.D190N	n/a	n/a
27	F	Caucasian	36	ADRP	RHO	c.404G>T	p.R135L	n/a	n/a
28	M	Caucasian	36	ADRP	RHO	c.266G>A	p.G89D	n/a	n/a
29	F	Caucasian	32	ADRP	RHO	c.68C>A	p.P23H	n/a	n/a
30	M	Caucasian	72	ADRP	RHO	c.316G>A	p.G106R	n/a	n/a
31	M	Caucasian	57	ADRP	RHO	c.800C>T	p.P267L	n/a	n/a
32	F	Caucasian	54	ADRP	RHO	c.1025G>A	p.T342M	n/a	n/a
33	M	Caucasian	40	ADRP	RHO	c.632A>C	p.H211P	n/a	n/a
34	F	Caucasian	27	ADRP	RHO	c.50C>T	p.T17M	n/a	n/a
35	F	Caucasian	43	ADRP	RHO	c.266G>A	p.G89D	n/a	n/a
36	F	Caucasian	34	ADRP	RHO	c.1040C>T	p.P347L	n/a	n/a
37	M	Hispanic	28	ADRP	RHO	c.1040C>T	p.P347L	n/a	n/a
38	M	Caucasian	46	ADRP	RHO	c.1040C>T	p.P347L	n/a	n/a
39	M	Hispanic	19	ADRP	RHO	c.50C>T	p.T17M	n/a	n/a
40	F	Hispanic	25	ADRP	RHO	c.50C>T	p.T17M	n/a	n/a
41	F	Caucasian	37	ADRP	RHO	c.541G>A	p.E181K	n/a	n/a
42	F	Caucasian	55	ADRP	RHO	c.83A>G	p.Q28R	n/a	n/a
43	F	Caucasian	53	ADRP	RP1	c.2285_2289del	p.L762YfsX17	n/a	n/a
44	M	Caucasian	23	ADRP	RP1	c.2105_2108del	p.I702TfsX10	n/a	n/a
45	M	Asian	46	ADRP	RP1	c.5017delT	p.Y1673MfsX37	n/a	n/a
46	M	Caucasian	65	ADRP	RP1	c.2285_2289del	p.L762YfsX17	n/a	n/a
47	F	Caucasian	44	ADRP	RP1	c.2479G>C	p.E827Q	n/a	n/a
48	M	Caucasian	30	ADRP	RP1	c.2029C>T	p.R677*	n/a	n/a
49	F	Afr.Am.	56	ADRP	RP1	c.2105_2108del	p.I702TfsX10	n/a	n/a
50	F	Caucasian	52	ADRP	RP1	c.2285_2289del	p.L762YfsX17	n/a	n/a
51	F	Caucasian	68	ADRP	RP1	c.2285_2289del	p.L762YfsX17	n/a	n/a
52	F	Asian	38	ADRP	RP1	c.1437G>T	p.M479I	n/a	n/a
53	M	Caucasian	55	ADRP	RP1	c.1234dupA	p.M412NfsX7	n/a	n/a

54	M	Caucasian	23	ADRP	RP1	c.5624G>C	p.G1875A	n/a	n/a
55	M	Caucasian	72	ADRP	SNRNP200	c.2041C>T	p.R681C	n/a	n/a
56	F	Caucasian	50	ADRP	SNRNP200	c.4487A>G	p.N1496S	n/a	n/a
57	F	Afr. Am.	71	ADRP	SNRNP200	c.4346A>G	p.N1449S	n/a	n/a
58	F	Caucasian	13	ARRP	C21ORF2	c.320A>G	p.Y107C	c.320A>G	p.Y107C
59	M	Caucasian	69	ARRP	CNGB1	c.2284C>T	p.R762C	c.2284C>T	p.R762C
60	F	Hispanic	28	ARRP	CNGB1	c.3150delG	p.F1051LfsX12	c. 3150delG	p.F1051LfsX12
61	F	Hispanic	60	ARRP	CNGB1	c.1896C>A	p.C632X	c.3150delG	p.G1050GfsX13
62	M	Caucasian	43	ARRP	CNGB1	c.2284C>T	p.R762C	c.2284C>T	p.R762C
63	F	Caucasian	32	ARRP	DHDDS	c.124A>G	p.K42E	c.124A>G	p.K42E
64	F	Caucasian	40	ARRP	DHDDS	c.124A>G	p.K42E	c.124A>G	p.K42E
65	M	Caucasian	69	ARRP	EYS	c.5928delG	p.R1976SfsX11	c.1308C>A	p.C436X
66	F	Caucasian	36	ARRP	EYS	c.6714del	p.I2239SfsX17	c.1299+5_1299+8del	p.?
67	F	Asian	33	ARRP	EYS	c.3555C>G	p.C1185W	c.8633C>G	p.A2878G
68	F	Caucasian	57	ARRP	EYS	c.6528C>A	p.Y2176X	c.6528C>A	p.Y2176X
69	M	Caucasian	29	ARRP	EYS	c.4120C>T	p.R1374X	c.4120C>T	p.R1374X
70	M	Caucasian	61	ARRP	EYS	c.6714del	p.I2239SerfsX17	c.9299_9302delCTCA	p.T3100KfsX26
71	M	Caucasian	56	ARRP	EYS	c.1645G>T	p.E549X	c.2992+1G>A	p.?
72	M	Hispanic	27	ARRP	EYS	c.7578+1G>A	p.?	c.7578+1G>A	p.?
73	M	Caucasian	57	ARRP	EYS	c.9383_9387del	p.?	c.6571+5G>A	p.?
74	M	Caucasian	66	ARRP	EYS	c.8648_8655del	p.T2883KfsX4	c.1155T>A	p.C385X
75	M	Caucasian	64	ARRP	EYS	c.8012T>A	p.L2671X	c.1641_1644delTCAG	p.?
76	M	Caucasian	47	ARRP	EYS	c.9317_9336del	p.Thr3106KfsX13	c.9004C>T	p.Q3002X
77	M	Hispanic	24	ARRP	FAM161A	c.733A>G	p.M254V	c.1309A>T	p.R437X
78	M	Caucasian	49	ARRP	FAM161A	c.1321dupC	p.H441PfsX15	c.1321dupC	p.H441PfsX15
79	M	Caucasian	56	ARRP	KIZ	c.119_122delAACT	p.K40IfsX14	c.119_122delAACT	p.K40IfsX14
80	M	Caucasian	60	ARRP	KIZ	c.226C>T	p.R76X	c.226C>T	p.R76X
81	M	Caucasian	21	ARRP	KIZ	c.226C>T	p.R76X	c.226C>T	p.R76X
82	M	Caucasian	44	ARRP	MAK	c.1297_1298insAlu	p.Lys433insAlu	c.1297_1298insAlu	p.Lys433insAlu
83	M	Caucasian	71	ARRP	MAK	c.1297_1298insAlu	p.Lys433insAlu	c.1297_1298insAlu	p.Lys433insAlu
84	M	Caucasian	35	ARRP	MAK	c.1297_1298insAlu	p.Lys433insAlu	c.1297_1298insAlu	p.Lys433insAlu
85	M	Caucasian	32	ARRP	MAK	c.1297_1298insAlu	p.Lys433insAlu	c.1297_1298insAlu	p.Lys433insAlu
86	F	Afr.Am.	22	ARRP	MERTK	Partial deletion at 2q13 (77kb)	p.?	Partial deletion at 2q13 (77kb)	p.?
87	F	Hispanic	12	ARRP	MERTK	c.2189+1G>T	p.?	c.2189+1G>T	p.?
88	F	Other	27	ARRP	PCDH21	c.2522_2528del	p.I841SfsX11		p.I841SfsX11
89	M	Hispanic	45	ARRP	PDE6A	c.304C>A	p.R102C	c.908C>G	p.S303C
90	M	Caucasian	14	ARRP	PDE6A	c.1705C>A	p.Q569K	c.2263C>T	p.Q755X
91	M	Hispanic	32	ARRP	PDE6A	c.304C>A	p.R102C	c.908C>G	p.S303C
92	M	Caucasian	15	ARRP	PDE6B	c.1923_1969ins6del47	p.?	c.1923_1969ins6del47	p.?
93	M	Caucasian	81	ARRP	PDE6B	c.1923_1969ins6del47	p.?	c.1923_1969ins6del47	p.?
94	F	Asian	57	ARRP	PDE6B	c.1488delC	p.T497PfsX78	c.1669C>T	p.H557Y
95	M	Hispanic	51	ARRP	PDE6B	c.1655G>A	p.R552Q	c.1655G>A	p.R552Q
96	F	Hispanic	47	ARRP	PDE6B	c.1540delC	p.?	c.1540delC	p.?
97	M	Caucasian	34	ARRP	PDE6B	c.1927_1967del41	p.?	c.1927_1967del41	p.?
98	M	Other	43	ARRP	PDE6B	c.756delC	p.D252EfsX29	c.2332G>A	p.V778M
99	F	Caucasian	50	ARRP	PROM1	c.223A>G	p.T75A	c.718G>A	p.G240R
100	M	Caucasian	31	ARRP	REEP6	c.295G>A	p.E99K	c.295G>A	p.E99K
101	F	Hispanic	59	ARRP	RP1	c.1510T>G	p.S504A	c.1510T>G	p.S504A
102	M	Other	12	ARRP	SCAPER	c.2023-2A>G	p.?	c.2023-2A>G	p.?
103	M	Caucasian	50	ARRP	SPATA7	c.1238A>G	p.H413R	c.1238A>G	p.H413R
104	M	Asian	26	ARRP	TULP1	c.349G>A	p.E117K	c.349G>A	p.E117K
105	F	Caucasian	46	ARRP	USH2A	c.10116T>A	p.N3372K	c.15519+5G>C	p.?
106	F	Caucasian	52	ARRP	USH2A	c.7524del	p.R2509GfsX19	c.14453C>T	p.P4818L
107	F	Hispanic	41	ARRP	USH2A	c.920_923dupGCCA	p.H308Qfs	c.10073G>A	p.C3358Y
108	M	Caucasian	75	ARRP	USH2A	c.7863delA	p.2623HfsX18	c.12575G>A	p.R4192H
109	M	Caucasian	50	ARRP	USH2A	c.2276G>T	p.C759F	c.12294+1G>A	p.?
110	M	Asian	69	ARRP	USH2A	c.13378A>T	p.I4460L	c.15178T>C	p.S5060P
111	M	Other	46	ARRP	USH2A	c.10073G>A	p.C3358Y	c.9119G>A	p.R3037H

112	M	Afr.Am.	57	ARRP	USH2A	c.478G>A	p.G160S	c.9959-11T>G	p.?
113	M	Caucasian	67	ARRP	USH2A	c.9371+3A>G	n/a	c.11048-2A>G	p.?
114	F	Caucasian	66	ARRP	USH2A	c.12575G>A	p.R4192H	c.12575G>A	p.R4192H
115	F	Caucasian	41	ARRP	USH2A	c.2299delG	p.E767SfsX21	c.2276G>T	p.C759F
116	F	Caucasian	29	ARRP	USH2A	c.9676C>T	p.R3226X	c.1478A>G	p.Y493C
117	M	Caucasian	20	ARRP	USH2A	c.4251+1G>A	p.?	c.13223T>C<br>c.13231C>G	p.V4408A<br>p.L4411V
118	M	Caucasian	67	ARRP	USH2A	c.2276G>T	p.C759F	c.2276G>T	p.C759F
119	F	Caucasian	30	ARRP	USH2A	c.10073G>A	p.C3358Y	c.10759C>T	p.Q3587X
120	F	Hispanic	24	ARRP	USH2A	c.9740-1G>T	p.?	c.13010C>T	p.T4337M
121	F	Hispanic	51	ARRP	USH2A	c.895delC	p.Q299NfsX37	c.5848A>G	p.T1950A
122	M	Other	49	ARRP	USH2A	c.13491_13499dup	p.T4498_T450dup	c.13491_13499dup	p.T4498_T450dup
123	M	Caucasian	55	ARRP	USH2A	c.12575G>A	p.R4192H	c.8682-9A>G	p.?
124	F	Hispanic	32	ARRP	USH2A	c.2276G>T	p.C759F	c.2276G>T	p.C759F
125	F	Caucasian	62	ARRP	USH2A	c.3368A>G	p.Y1123C	c.12575G>A	p.R4192H
126	F	Afr.Am.	31	ARRP	USH2A	c.10490A>G	p.D3497G	c.13507G>A	p.V4503M
127	M	Hispanic	93	ARRP	USH2A	c.6931del	p.A231PfsX15	c.6937G>T	p.G231C
128	M	Other	44	ARRP	USH2A	c.457T>A	p.W153R	c.10342G>A	p.E3448K
129	M	Caucasian	28	ARRP	USH2A	c.12575G>A	p.R4192H	c.2802T>G	p.C934W
130	M	Caucasian	63	ARRP	USH2A	c.12575G>A	p.R4192H	c.7595-2144A>G	p.?
131	F	Asian	37	ARRP	USH2A	c.99_100insT	p.R34SfsX41	c.5497G>A	p.V1833M
132	M	Caucasian	30	ARRP	USH2A	c.11864G>A	p.W3955X	c.12575G>A	p.R4192H
133	M	Hispanic	61	ARRP	USH2A	c.1246G>A	p.A416T	c.12575G>A	p.R4192H
134	M	Caucasian	68	ARRP	USH2A	c.3684T>A	p.Cys1228X	c.6937G>T	p.G2313C
135	M	Caucasian	39	ARRP	USH2A	c.12343C>T	p.R4115C	c.13274C>T	p.T4425M
136	F	Caucasian	12	USH1	PCDH15	c.733C>T	p.R245X	c.733C>T	p.R245X
137	M	Hispanic	12	USH1	MYO7A	c.4544_4551del<br>AGATCAGinsCA	p.?	c.4544_4551del<br>AGATCATGinsCA	p.?
138	M	Hispanic	7	USH1	MYO7A	c.634C>T	p.R212C	c.999T>G	p.Y333X
139	F	Hispanic	18	USH1	MYO7A	c.999T>G	p.Y333X	c.999T>G	p.Y333X
140	F	Other	12	USH1	MYO7A	c.5581C>T	p.R1861X	c.5507T>C	p.L1836P
141	F	Hispanic	16	USH1	MYO7A	c.3508G>A	p.E1170K	c.6070C>T	p.R2024X
142	F	Caucasian	41	USH2	GPR98	c.9430G>T	p.E3144X	c.10769+2T>A	p.?
143	M	Caucasian	24	USH2	GPR98	c.15520-22_15524del27	p.?	c.6049+2T>G	p.?
144	M	Hispanic	24	USH2	USH2A	c.1724G>A	p.C575Y	c.2299delG	p.E767fs
145	M	Caucasian	21	USH2	USH2A	c.3713C>G	p.T1238R	c.9459C>A	p.C3153X
146	M	Afr.Am.	45	USH2	USH2A	c.1000C>T	p.R334W	c.8442_8443insT	p.?
147	F	Caucasian	24	USH2	USH2A	c.10712C>T	p.T3571M	c.11918delC	p.?
148	F	Caucasian	27	USH2	USH2A	c.2299delG	p.?	c.2299delG	p.?
149	F	Caucasian	23	USH2	USH2A	c.13112_13115delAAAT	p.?	c.13943delG	p.?
150	F	Caucasian	35	USH2	USH2A	c.10712C>T	p.T3571M	c.4711G>C	p.A1571P
151	M	Hispanic	18	USH2	USH2A	c.15520-22_15524del27	p.?	c.6049+2T>G	p.?
152	M	Other	29	USH2	USH2A	c.13577G>A	p.R4526Q	c.5775A>G	p.T1925T
153	M	Caucasian	28	USH2	USH2A	c.2299del	p.E767SfsX21	c.5603T>G	p.F1868C
154	M	Afr.Am.	62	USH2	USH2A	c.11506C>T	p.P3836S	c.274T>G	p.S92A
155	F	Caucasian	10	USH3	CLRN1	c.144T>G	p.N48K	c.144T>G	p.N48K
156	M	Caucasian	8	USH3	CLRN1	c.144T>G	p.N48K	c.144T>G	p.N48K
157	F	Hispanic	15	BBS	BBS1, BBS9	c.1645G>T	p.E549X	c.1281G>A	p.A427A
158	M	Other	12	XLRP	RPGR	c.202G>A	p.G68R	n/a	n/a
159	M	Caucasian	30	XLRP	RPGR	c.2194del	p.E732RfsX83	n/a	n/a
160	M	Caucasian	13	XLRP	RPGR	c.1307G>A	p.G436D	n/a	n/a
161	M	Caucasian	6	XLRP	RPGR	c.454T>C	p.S152P	n/a	n/a
162	M	Hispanic	33	XLRP	RPGR	c.2571delA	p.?	n/a	n/a
163	M	Caucasian	16	XLRP	RPGR	c.1059_1059+2delGGT	p.?	n/a	n/a
164	M	Hispanic	43	XLRP	RPGR	c.3027_3028del	p.E1010GfsX68	n/a	n/a
165	M	Other	36	XLRP	RPGR	c.2426_2427del	p.E809GfsX25	n/a	n/a
166	M	Caucasian	7	XLRP	RPGR	c.2966delA	p.?	n/a	n/a
167	M	Caucasian	53	XLRP	RPGR	c.2155delC	p.H719Ifs	n/a	n/a
168	M	Hispanic	17	XLRP	RPGR	c.2323_2324delAG	p.R775Efs	n/a	n/a

* Age at presentation. Afr.Am. = African American ; ADRP = autosomal dominant retinitis pigmentosa; ARRP = autosomal recessive retinitis pigmentosa; BBS = Bardet-Biedl syndrome; F= female; M = male; n/a = not applicable; USH1 = Usher Syndrome type 1; Usher Syndrome type 2; USH3 = Usher Syndrome type 3; XLRP = X-linked retinitis pigmentosa.
